# Design, Validation, and Reliability of an Observational Instrument for Technical and Tactical Actions in Singles Badminton

**DOI:** 10.3389/fpsyg.2020.582693

**Published:** 2020-12-10

**Authors:** Gema Torres-Luque, Juan Carlos Blanca-Torres, José María Giménez-Egido, David Cabello-Manrique, Enrique Ortega-Toro

**Affiliations:** ^1^Faculty of Humanities and Education Sciences, University of Jaén, Jaén, Spain; ^2^Sports Performance Analysis Association, Murcia, Spain; ^3^Faculty of Sport Science, Campus Mare Nostrum, University of Murcia, Murcia, Spain; ^4^Department of Physical Education and Sports, Sport Science Faculty, University of Granada, Granada, Spain

**Keywords:** racket sports, badminton, test, performance analysis, observational methodology

## Abstract

Technical and tactical actions are decisive in terms of badminton player competitive performance. The main objective of this research was to design, validate, and estimate the reliability of an observational instrument for the analysis of the tactical and technical actions in individual badminton. The process was carried out in four different steps: first, there was a review of the scientific literature and a preliminary list of variables was made; second, a qualitative and quantitative assessment was completed by 10 badminton expert judges; in the third step, the content validity was estimated using Aiken’s V coefficient; finally, intra-observer reliability and interobserver reliability were tested by two observers specialized in badminton using the Cohen’s Kappa coefficient and the intraclass correlation coefficient. Strokes were used as the unit of measure by our observational instrument; every time badminton players hit the shuttlecock, 22 variables (eight contextual variables, seven variables related to the result of the match, and seven variables related to the game) are observed. The minimum Aiken’s value was 0.58, and reliability was 0.63. In spite of these values, none of the variables had to be removed, but there were modifications in terms of drafting in some of them. The main findings confirmed the validity and the usefulness of this instrument.

## Introduction

Nowadays, the observational methodology has become an essential tool that allows coaches to improve their training programs, athletes to make better technical tactical decisions, sports organizations to manage teams more effectively, and academic researchers to develop a better understanding of sports performance (O’Donoghue, 2014). With the objective of guaranteeing the quality of research, the observational methodology needs to validate reliable procedures with respect to the design of variables and categories (Lago Peñas et al., 2020). This requires the participation of coaches, experts, and observers in the entire validation process (Ortega et al., 2008a; Moreno and Gómez, 2017).

Different methods are used by sports sciences to carry out this type of studies (Figueira et al., 2018). In recent years, in spite of the technological advances (Mateus et al., 2017), the observational methodology (Anguera et al., 2011; Maneiro et al., 2018; Fernández-García et al., 2020) has been used by researchers to extract valuable information about sport actions, since it allows to know different actions that are executed during the game time, as well as the contexts where they occur. Likewise, it is a method that requires rigor at the time of observational instrument design and in the process of observation and data analysis (Gorospe et al., 2005; Valldecabres et al., 2019).

In the sports field, studies about validating a specific observational instrument have been increasing. Some of these have been carried out in physical education (Ortega et al., 2008a,b) and some team sports such as soccer (Fernandes et al., 2019; Ortega-Toro et al., 2019), basketball (Ibáñez et al., 2019), handball (Morillo et al., 2017), rugby (Villarejo et al., 2014), and volleyball (Palao et al., 2015a). Studies of these characteristics have also been carried out in individual sports like judo (Rodríguez et al., 2016) and, in particular, in other racket sports, as well as in tennis (Gorospe et al., 2005; Torres-Luque et al., 2018) or table tennis (Pradas et al., 2012). Depending on each investigation, the procedure is described in different steps: to review the scientific literature; to design the instrument with the system of conducts and categories to be observed; to evaluate the instrument quantitatively and qualitatively; to calculate the content validity of the instrument; to test the reliability.

In badminton, which is characterized by high-speed movements and shot executions (Abdullahi and Coetzee, 2017), observational methodology has been used to obtain information about different game aspects (Abián et al., 2014; Abdullahi and Coetzee, 2017), but the rules and characteristics of this sport make it difficult to observe and analyze the game. In this sense, previous studies carried out in similar disciplines such as squash (Brown and Hughes, 1995) or table tennis (Pradas et al., 2012) have been taken into account, whose observational instruments have been created *ad hoc* in order to solve this problem.

Therefore, it is important to explain the design and validation process of the instrument that is used to collect this information. Thus, the main goal of this study is to design and validate an observational instrument for researchers and badminton coaches to assess the technical tactical motions in singles badminton with reliability, objectivity, accuracy, and validity.

## Materials and Methods

The design, validation, and testing of the reliability of the badminton observational instrument was completed in four stages. The first consisted of the observation instrument design that was a mixed category system and field format (Anguera and Hernández-Mendo, 2015; Anguera et al., 2018); the established categories were exhaustive and mutually exclusive (E/ME). The second consisted of the quantitative and qualitative evaluation of the instrument. The third consisted of the validation and calculation of the content. The fourth consisted of a reliability test (Kinrade et al., 2010).

Preparing a draft list of the conducts to be studied after a scientific literature review, mainly in the Web of Science databases with the keyword “badminton” (Tables 1–3), was the purpose of the first stage. The result showed a first list of variables with their definitions and categories to which they should belong. Three groups of variables were established: (a) contextual variables, including all those that defined the conditions of a match (gender, competition level, type of event round, game mode, court surface, shuttlecock type, and player laterality). The analysis unit was the match; (b) variables related to the result of the match, including all score statistics (match winner or loser, analyzed set, sets in favor, sets against, winner or loser of the analyzed set, game scoring, and analyzed point loser or winner). The unit of analysis was the match; (c) variables related to the game (stroke sequence, point duration time, type of technical and tactical stroke, trajectory, tactical intentionality, hitting area, and stroke effectiveness). The unit of analysis was the point.

**TABLE 1:** List of contextual variables, variables related to the result of the match, and variables related to the game and their respective categories that make up the observational instrument after the first phase.

Contextual variables	Categories	
Gender of the players*	• Male	• Mixed
	• Female	
Competition level*	• Professional badminton	• Provincial badminton
	• Semi-Professional badminton	• Amateur badminton
	• National badminton	• Others
	• Regional badminton	
Type of tournament*	• Olympic Games	• Spanish Championship
	• World Cup	• National competitions
	• European Championship	• Regional competitions
	• Open	• Provincial competitions
	• Master 1000	• Local competitions
	• Master 500	• Non-federated championships
	• Master 300	• Others
Tournament round*	• League phase	• Round of sixteen
	• Final	• Sixteenths
	• Semifinals	• Treintaydosavos
	• Quarter finals	•
Game mode*	• Best of 3 sets of 21 points with a	• Best of 1 set of 21 points with a
	• difference of 2 up to a limit of 30	• difference of 2 up to a limit of 30
	• Best of 3 sets of 15 points with a difference of 2 up to a limit of 21	• Best of 1 set of 15 points with a difference of 2 up to a limit of 21
	• Best of 3 sets of 11 points with a difference of 2 up to a limit…	• Best of 1 set of 11 points with a difference of 2 up to a limit…
Court surface*	• Carpet	• Rubber
	• Parquet	• Others
Shuttlecock type*	• Natural	• Synthetic
Laterality of the players*	• Right handed	• Left handed

**Variables related to the result of the match**	**Categories**	

Winner or loser of the match*	• Winner	• Loser
Analyzed set*	• 1st set	• 3rd set
	• 2nd set	
Sets in favor*	• No set in favor	• A set in favor
Sets against*	• No set against	• A set against
Winner or loser of the analyzed set*	• Winner of the set	• Loser of the set
Game score*	• 0/0	• 0/1
	• 1/0	• …
Winner or loser of the analyzed point*	• Winner of the point	• Loser of the point

**Variables related to the game**	**Categories**	

Stroke sequence^∗^	• Serve • Return • 2nd stroke of the point, 3rd stroke of the point…	• Penultimate stroke of the point • Last stroke of the point
Point duration time^∗^		
Kind of technical and tactical stroke^∗^	• Right serve • Reverse serve • Offensive serve • Defensive serve • High serve • Flick serve • Drive serve • Short serve • Clear from right to high hand • Clear from right to medium height	• Right hand drive • Left hand drive • Drive to high hand • Drive to medium height • Parallel drive • Cross drive • Right net drop • Left net drop • Net drop to medium height • Net drop to low hand
	• Clear from right to low hand • Clear from left to high hand • Clear from left to medium height • Clear from left to low hand • Offensive clear • Defensive clear • Parallel clear • Cross clear • Right drop • Left drop • Drop to high hand • Drop to medium height • Drop to low hand • Parallel drop • Cross drop • Slow drop • Fast drop • Drop shot • Reverse drop • Right smash • Left smash • Parallel smash • Cross smash • Smash in jump	• Offensive net drop • Defensive net drop • Parallel net drop • Cross net drop • Right lob • Left lob • Lob to medium height • Lob to low hand • Offensive lob • Defensive lob • Parallel lob • Cross lob • Right brush • Left brush • Right kill • Left kill • Parallel kill • Cross kill • Right push • Left push • Parallel push • Cross push
Trajectory^∗^	• Parallel	• Cross
Tactical Intentionality^∗^	• Offensive	• Defensive
Hitting area^∗^	• Inside the court, serve and background zone, in the right area • Inside the court, serve and background zone, in the central area • Inside the court, serve and background zone, in the left area • Serve zone in the right area	• Serve zone in the central area • Serve zone in the left area • Near the net in the right area • Near the net in the central area • Near the net in the left area
Stroke effectiveness^∗^	• Winner • Total continuity	• Partial continuity • Error

*Suggested behaviors in the review of the scientific literature (first phase).

**TABLE 2:** Sample questionnaire sent to the 10 badminton expert judges.

**Stroke effectiveness**
**Variable:** effectiveness of the stroke performed by the player.
**Categories:** **1. Winner.** Stroke performed by the player with the one that gets the point directly, without his/her opponent touched the shuttlecock. **2. Total continuity.** Transitional stroke performed by the player, who sends the shuttlecock to the opposite field continuing his/her rival the point (without failure). **3. Partial continuity.** Stroke performed by the player, who sends the shuttlecock to the opposite field, causing his/her rival to hit the shuttlecock sending it out of the regulatory area of the court, to the net, to the roof or to their own field (with failure). **4. Error.** Stroke performed by the player, who sends the shuttlecock out of the regulatory area of the court, to the net, to the roof or to their own field, losing the point.
**(a) Inclusion:** Do you consider it necessary to include this variable in the observation sheet? YES/NO
**(b) Adequacy:** Do you think that the definition of the variable and its categories is adequate? • • Very inadequate 1-2-3-4-5-6-7-8-9-10 Very suitable •
**Drafting:** Do you consider adequate the wording of the definition of the variable and the definition of each of the categories? • • Very poorly drafted 1-2-3-4-5-6-7-8-9-10 Very well drafted
**Observations:**
*Variable stroke effectiveness*

**TABLE 3:** Final list of contextual variables, variables related to the result of the match, variables related to the game and their respective categories that make up the observational instrument.

Contextual variables	Categories	
Gender of the players*	• Male • Female	• Mixed
Tournament level*	• Professional badminton • Semi-Professional badminton • National badminton • Regional badminton	• Provincial badminton • Amateur badminton • Others
Type of tournament**	• Olympic Games • World Cup • European Championship • Open • Master 1000 • Master 500 • Master 300	• Spanish Championship • National competitions • Regional competitions • Provincial competitions • Local competitions • Non-federated championships • Others
Tournament round*	• League phase • Final • Semifinals • Quarter finals	• Round of sixteen • Sixteenths • Treintaydosavos
Game mode*	• Best of 3 sets of 21 points with a difference of 2 up to a limit of 30 • Best of 3 sets of 15 points with a difference of 2 up to a limit of 21 • Best of 3 sets of 11 points with a difference of 2 up to a limit…	• Best of 1 set of 21 points with a difference of 2 up to a limit of 30 • Best of 1 set of 15 points with a difference of 2 up to a limit of 21 • Best of 1 set of 11 points with a difference of 2 up to a limit…
Court surface**	• Carpet • Parquet	• Rubber • Others
Shuttlecock type*	• Natural	• Synthetic
Laterality of the players*	• Right handed	• Left handed

**Variables related to the result of the match**	**Categories**	

Winner or loser of the match*	• Winner	• Loser
Analyzed set*	• 1st set • 2nd set	• 3rd set
Sets in favor*	• No set in favor	• A set in favor
Sets against*	• No set against	• A set against
Winner or loser of the analyzed set*	• Winner of the set	• Loser of the set
Game score*	• 0/0 • 1/0	• 0/1 • …
Winner or loser of the analyzed point*	• Winner of the point	• Loser of the point

**Variables related to the game**	**Categories**	

Stroke sequence**	• Serve error • Serve • 2nd stroke of the point, 3rd stroke of the point…	• Penultimate stroke of the point • Last stroke of the point
Point duration time*		
Kind of technical and tactical stroke*	• Right serve • Reverse serve • Clear from right to high hand • Clear from right to medium height • Clear from right to low hand • Clear from left to high hand • Clear from left to medium height • Clear from left to low hand • Right drop • Left drop • Right smash • Left smash • Smash in jump • Drive from right to high hand	• Lob from right to medium height • Lob from right to low hand • Lob from left to medium height • Lob from left to low hand • Right brush • Left brush • Right kill • Left kill • Right push • Left push
	• Drive from right to medium height • Drive from left to high hand • Drive from left to medium height • Net drop from right to medium height • Net drop from right to low hand • Net drop from left to medium height • Net drop from left to low hand	
Trajectory*	• Parallel	• Cross
Tactical intentionality*	• Offensive	• Defensive
Hitting area*	• Inside the court, serve and background zone, in the right area • Inside the court, serve and background zone, in the central area • Inside the court, serve and background zone, in the left area • Serve zone in the right area	• Serve zone in the central area • Serve zone in the left area • Near the net in the right area • Near the net in the central area • Near the net in the left area
Stroke effectiveness**	• Winner • Total continuity	• Partial continuity • Error

*Behaviors selected after the first phase; ^∗∗^Behaviors with modifications suggested by experts (third phase).

Table 2 presented a sample questionnaire that was sent to the 10 badminton expert judges with a variable “stroke effectiveness” as an example.

Table 1 showed a preliminary list of contextual variables, variables related to the result of the match, and variables related to the game and their respective categories with suggested behaviors in the review of scientific literature that make up the observational instrument.

In the second stage, a qualitative and quantitative assessment of the observation instrument was completed by 10 badminton expert judges with the minimum following requirements: (a) Sports Technician certificate on the highest national level; (b) an experience of more than 10 years in teaching badminton; (c) Master in Sport Sciences or Physical Activity. For the assessment, the experts completed a survey (Table 4) with the following questions about the variable: (a) level of interest to include the variable (inclusion); (b) level of adequacy in the variable and categories definition (adequacy); (c) level of drafting of the variable and categories definition (drafting); (d) the quantitative assessment was to score from 1 to 10 the adequacy and drafting sections, while the qualitative part of the assessment consisted of responding to the inclusion section “Yes” or “No” (observation). Therefore, the answers were made individually, including a quantitative and qualitative part. All data were registered, and a descriptive analysis was completed (average, median, and mode of every continuous variable and also relative and absolute frequency in the case of categorical variables).

**TABLE 4:** Values of pertinence, definition (Aiken’s V coefficient), and interobserver and intra-observer reliability (Cohen’s Kappa and ICC) of definitive variables and categories of the badminton observational instrument.

Variables	Pertinence (V Aiken)	Definition (V Aiken)	Intra-observer 1 Reliability (Cohen’s kappa)	Intra-observer 2 Reliability (Cohen’s kappa)	Interobserver reliability (Cohen’s kappa)
**Contextual**					
Gender of the players	0.89	0.89	1	1	1
Tournament level	0.81	0.81	1	1	1
Type of tournament	0.78	0.69	1	1	1
Tournament round	0.81	0.85	1	1	1
Game mode	0.85	0.86	1	1	1
Court surface	0.58	0.69	1	1	1
Shuttlecock type	0.79	0.82	1	1	1
Laterality of the players	0.86	0.86	1	1	1
**Result**					
Winner or loser of the match	0.72	0.67	1	1	1
Analyzed set	0.83	0.87	1	1	1
Sets in favor	0.86	0.90	1	1	1
Sets against	0.75	0.79	1	1	1
Winner or loser of the analyzed set	0.77	0.77	1	1	1
Game scoreboard	0.89	0.84	1	1	1
Winner or loser of the analyzed point	0.86	0.79	1	1	1
**Game**					
Stroke sequence	0.86	0.65	1	1	1
Point duration time	0.77	0.73	1 (ICC)	0.87 (ICC)	
Kind of technical and tactical stroke	0.82	0.60	0.91	1	0.87
Trajectory	0.90	0.77	1	1	0.94
Tactical intentionality	0.84	0.69	0.86	1	0.63
Hitting area	0.88	0.76	1	0.92	0.96
Stroke effectiveness	0.90	0.66	1	1	1

**ICC, intraclass correlation coefficient*.*

The third stage aim was to calculate the content validity through Aiken’s V coefficient (Aiken, 1980; Penfield and Giacobbi, 2004). Using the Visual Basic 6.0 software application described by Soto and Segovia (2009), Aiken’s V coefficient (Aiken, 1985) was used to determine the criteria for the modification or elimination of the variables. The obtained data were 0.70 (*p* = 0.05) and 0.81 (*p* = 0.01). Then, as a critical level of Aiken’s V was determined to reject the null hypothesis, it was decided to remove the variables whose data were lower than 0.70 and to modify the variables whose data were between 0.70 and 0.81. The variables data higher than 0.81 were accepted. In the same way, a minimum inclusion value of at least 80% “Yes” from the expert judges was accepted for the inclusion section. After those modifications, the next step was to create a new and definitive observation manual with a list of variables and categories that take to the writing of the observation instrument, which included the variables with the grouped categories of each one, their definitions and coding (“Observation instrument for singles badminton,” see Supplementary Annex 1).

In the fourth stage, the observation instrument reliability was tested following other studies (Gamonales et al., 2018; Torres-Luque et al., 2018). According to the studies by Anguera (2003) and Losada-López and Manolov (2015), three observers were trained by the main research supervisor through three sessions of 2 h each with a break of 10 min once they reached 55 from the observation using the fourth stage of the designed observation manual. In this case, to evaluate the reliability, two observers, both of them with masters in Primary Education with a mention in Physical Education and specialized in badminton, carried out the assessment twice within a week, 30 strokes of one badminton match. For the inter-observer and intra-observer reliability of all variables, a Cohen’s Kappa coefficient was used, except for the variable “duration of the point,” in which the intraclass correlation coefficient (ICC) was used.

As a last resort, it is very important and necessary to work out the specific protocol for the correct use of the observational instrument. Firstly, the observers must be in an elevated position behind a baseline of the badminton court, providing a good view of all lines (at least at the height of the players). The observational data collection in relation to the service area, hitting area, and shuttlecock end area was carried out according to the zones described in the observation manual.

The data were collected in an Excel-designed spreadsheet, where each row corresponds to a stroke of a player (each unit of measurement is a stroke by one player). The main reason of this recording is to understand the sequence of strokes in the interaction between players. For the purpose of optimizing the recording time of all variables and their categories, was defined as follows: (a) variables to record every stroke [results (game scoring and loser or winner of the analyzed point) and game development (stroke sequence, kind of technical-tactical stroke, hitting area, trajectory and stroke effectiveness)]; (b) variables to register every point [results (lost and won points on the set)]; (c) variables to register every set [results (analyzed set, lost and won sets, and loser and winner of the analyzed set)]; (d) variables to analyze every match [contextual (gender, event level, type of event, event round, game mode, court surface, type of shuttlecock, and player laterality) and results (loser or winner of the match)].

## Results

Table 1 shows the results of the observational instrument design after the scientific literature review. All game variables were determined, thanks to the researchers’ input and previous researches, and they were used as a basis to design the observational instrument. Finishing the first phase, 22 variables formed the observational instrument: eight contextual variables, seven variables related to the result of the match, and seven variables related to the game.

After performing the qualitative and quantitative assessment of the observation instrument by a group of experts (*n* = 10) and calculating the content validity using the Aiken’s V coefficient (Penfield and Giacobbi, 2004), a total of five modifications were carried out in the defined ones, two related to contextual variables and three related to game variables. All changes were endorsed with a lower Aiken’s V value and also when more than 80% of the expert judges respond positively to be included.

As can be seen in Table 3, there were not many changes after the evaluation, the main changes were found in the following variables: (a) court surface, the experts did not consider it necessary; (b) type of tournament, the experts proposed a change in nomenclature or join this variable with the competition level; (c) stroke sequence, the experts proposed to analyze a determined number of strokes; (d) tactical intentionality, the experts considered important to add more intentions (neutral intent, for example); (e) stroke effectiveness, the experts proposed to redefine the concepts due to their inaccuracy.

Table 3 showed a definitive list of contextual variables, variables related to the result of the match and variables related to the game and their respective categories that make up the observation instrument.

The 22 final variables were eight contextual variables, seven variables related to the result of the match, and seven variables related to the game (Table 3). Then, all variables with a value ≥ 0.81 in the Aiken’s V were considered for the observation instrument (Table 4). One hundred percent of the expert judges responded positively for the inclusion of all cases.

The values of pertinence, definition, and interobserver and intra-observer reliability of definitive variables and categories of the badminton observational instrument are shown in Table 4.

Table 4 showed the results from the fourth phase, which indicated high reliability values in general. As it can be observed, the variable “tactical intentionality” obtained the lowest value in Cohen’s Kappa coefficient, both in the intra-observer test (0.86) and in the interobserver test (0.63). The ICC was used for the variable “point duration time” with values of 1 and 0.87.

## Discussion

The present study has been carried out in order to show and explain all the stages for designing, validating, and testing the reliability of the observational instrument that analyzes the technical and tactical behaviors in singles badminton. Therefore, the designed observational instrument is valid and reliable to analyze the technical and tactical actions that take place during a rally in a badminton match. To this effect, the methodological procedures suggested by Anguera and Hernández-Mendo (2013) have been followed, just as other similar studies that have been carried out in different sports (Pradas et al., 2012; Villarejo et al., 2014; Palao et al., 2015a,b; Torres-Luque et al., 2018; Parada and Vargas, 2020). By this way, a valid and reliable tool has been generated for this sport.

A total number of 10 expert judges and two observers have participated in the design and validation of the observational instrument. In this respect, it should be noted that the particularity of the subject matter limits the existence of the experts in this area of knowledge. In spite of this, the number of experts was the same or higher than in other studies carried out in singles sports such as judo (Rodríguez et al., 2016) or table tennis (Pradas et al., 2012) and in other investigations done in collective sports like soccer (Parada and Vargas, 2020) or beach volleyball (Palao et al., 2015b).

The high qualification of the different expert judges, who follow the three criteria of inclusion, master in Physical Activity and Sports Sciences, with a coach diploma, and more than 10 years of training experience, should be noted. This level of qualification has provided the theoretical, together with their badminton experience, giving valuable information to researchers (Escobar-Pérez and Cuervo-Martínez, 2008; Gamonales et al., 2018; Ibáñez et al., 2019). Their qualitative and quantitative input was crucial for the design of the observational instrument and, thanks to them, the tool has been improved because some variables could be modified to clarify the definitions and their relevance to the different categories. The two observers have also been very important throughout the process, helping to define the criteria for the different categories and as a consequence simplifying the registration instrument.

In terms of statistics, the selected variables give an adequate content validity to the observational instrument, being valid to assess the technical and tactical actions in singles badminton, since the Aiken’s V value shows a positive evaluation of the different variables both in pertinence and in definition (Zartha et al., 2018). All values that have been obtained in this study outweigh the critical level proposed by Aiken (1985) or Penfield and Giacobbi (2004), who considered that from 0.50, the validity of the instrument can be accepted. In any case, most of the variables are higher than the critical level of 0.70, as proposed by Charter (2003) or Soto and Segovia (2009). Hence, it can be said that the results obtained show that the design of the variables of the observational instrument has indicators of content validity, since they are above the exact critical level proposed by experts.

The third stage of this research, as well as in the study carried out by Gamonales et al. (2018), consisted of modifying or eliminating those items that did not reach optimal Aiken’s V coefficient values according to the criteria proposed (García Martín et al., 2016). On the one hand, the quantitative assessment carried out by the experts was very fruitful to establish the variables and their categories (Bulger and Housner, 2007; Padilla et al., 2007). On the other hand, the qualitative assessment carried out by the experts was very fruitful to define the variables and their categories. In this study, despite the fact that expert judges had made contributions to improve the drafting of some variables, it had not been necessary to delete any of them.

With respect to reliability, the observation manual and the observers’ training helped them to get the skills for carrying out the observation (Losada-López and Manolov, 2015), increasing the effectiveness of the observation and improving the coding criteria. It should be emphasized that the intra-observer analysis evidences high levels of reliability, minimizing the observation mistakes that may come from the observer himself (Gamonales et al., 2018), but the different interpretations of the behaviors among the observers can cause disagreements (Losada-López and Manolov, 2015). Nevertheless, the interobserver analysis evidences high levels of reliability too, supporting the reliability of the observation (Liu et al., 2013).

The design of this observational instrument has some limitations, as the number of expert judges who have participated or the recording of some data related to the game development or physical components that may be interesting. In accordance with Lebed (2006), behaviors in sports are affected by an undermining number of factors. In badminton, the specificity of the players’ behaviors, together with the high speed, makes the recording difficult, as in the case in other sports such as table tennis (Pradas et al., 2012). Nevertheless, it is complex to use collection information systems for evaluating players’ performance in competition (Villarejo et al., 2014). With the aim of improving, future studies should take into account these aspects when designing observational instruments.

## Conclusion

Therefore, the designed observational instrument is valid and reliable to analyze the technical and tactical actions that take place during a rally in a badminton match. Thereby, it is possible to analyze the differences between winners and losers and establish relationships between strokes during the sequence, which can affect the development and result of the point.

## Data Availability Statement

The raw data supporting the conclusions of this article will be made available by the authors, without undue reservation.

## Ethics Statement

The studies involving human participants were reviewed and approved by the University of Jaen (JUN.18/10.TES). The patients/participants provided their written informed consent to participate in this study.

## Author Contributions

GT-L and EO-T: conceptualization. JB-T, JG-E, and EO-T: methodology. GT-L, JB-T, JG-E, DC-M, and EO-T: formal analysis. JB-T, JG-E, and DC-M: investigation. GT-L, JB-T, JG-E, and EO-T: writing—original draft preparation. JG-E, EO-T, and GT-L: writing—review and editing. EO-T and DC-M: supervision. GT-L and EO-T: funding acquisition. All authors have read and agreed to the published version of the manuscript.

## Conflict of Interest

The authors declare that the research was conducted in the absence of any commercial or financial relationships that could be construed as a potential conflict of interest.
