# Supplementary material for: Design, Validation, and Reliability of an Observational Instrument for Technical and Tactical Actions in Singles Badminton
Source: Front Psychol. 2020 Dec 10;11:582693. doi: 10.3389/fpsyg.2020.582693 (PMC7758221; doi:10.3389/fpsyg.2020.582693)
Supplement: Supplementary file 1 [file Data_Sheet_1.PDF]

ANNEX 1

Observation instrument for singles badminton

This instrument uses the stroke as a unit of measure; so that each time a player hits a shuttlecock the following variables are analyzed:

- **Contextual variables.**
- **Variables related to the result of the match.**
- **Variables related to the game.**

1. CONTEXTUAL VARIABLES

The contextual variables provide information about the context in which the match is played. These variables will be fixed during the match

1.1 <u>Gender of the players:</u> the gender of the players will be specified. In the case of badminton, since there are no mixed competitions except in the case of doubles, the entire column of the variable will carry the same code, both for the winner and the loser of the match.

1	Male
2	Femal
3	Mixed

1.2 <u>Tournament level:</u> depending on the degree of professionalism of the tournament, several levels are established:

1	Professional badminton
2	Semi-professional badminton
3	National badminton
4	Regional badminton
5	Provincial badminton
6	Amateur badminton
7	Others

1 ***Professional badminton***
2 ***Semi-professional badminton***
3 ***National badminton:*** national championships of all categories.
4 ***Regional badminton:*** regional championships of all categories.
5 ***Provincial badminton****:* provincial championships of all categories.

*6 **Amateur badminton:*** federated competitions of any category, except national, regional or provincial championships.
*7 **Others:*** any other type of competition that is not within the above.

1.3 **Type of tournament:** within each of the different degrees of professionalism that tournaments may have, there are different tournament levels. In the case of professional tournaments, their level is directly proportional to the money distributed in prizes.

1	Olympic Games
2	World Cup
3	European Championship
4	Open
5	Master 1000
6	Master 500
7	Master 300
8	Spanish Championship
9	National competitions
10	Regional competitions
11	Provincial competitions
12	Local competitions
13	Non federated championships
14	Others

***14 Others:** *all tournaments which do not appear in the list.*

1.4 **Tournament round:** it will be specified in which phase of the tournament the analyzed match is.

0	League phase
1	Final
2	Semifinals
4	Quarter finals
8	Round of sixteen
16	Sixteenths
32	Treintaydosavos

1.5 **Game mode:** the number of sets to which the tournament matches are played will be specified.

1	Best of 3 sets of 21 points with a difference of 2 up to a limit of 30
2	Best of 3 sets of 15 points with a difference of 2 up to a limit of 21
3	Best of 3 sets of 11 points with a difference of 2 up to a limit…
4	Best of 1 set of 21 points with a difference of 2 up to a limit of 30
5	Best of 1 set of 15 points with a difference of 2 up to a limit of 21
6	Best of 1 set of 11 points with a difference of 2 up to a limit…

1.6 **Court surface:** the court surface will be register.

1	Carpet
2	Parquet
3	Rubber
4	Others

4 **Others: all surfaces which do not appear in the list.*

1.7 **Shuttlecock type:** the type of shuttlecock will be register.

1	Natural
2	Synthetic

1.8 **Laterality of the players:** the player's dominant arm will be register.

1	Right handed
2	Left handed

2. VARIABLES RELATED TO THE RESULT OF THE MATCH

The variables related to the result of the match provide information on the momentary score of the match or its final result. The purpose of their registration is twofold, on the one hand, to check the influence of the result on what happens in the game and, on the other hand, to check the possible differences that may exist in the other variables between the winning and losing player.

2.1 **Winner or loser of the match:** it will be constantly recorded if the player analyzed is the winner or loser of a match. The first row of each one of the points analyzed will always have the code 1, corresponding to the winner and the second one the 0, corresponding to the loser.

1	Winner
0	Loser

2.2 **Analyzed set:** number of set which is being analyzing within the match.

1	1st set
2	2nd set
3	3rd set

2.3 **Sets in favor:** number of sets which the player has won in the match at the time of analysis of a specific point.

0	No set in favor
1	A set in favor

2.4 **Sets against:** number of sets which the player has lost in the match at the time of analysis of a specific point.

0	No set against
1	A set against

2.5 **Winner or loser of the analyzed set:** this variable will be constantily noted if the player being analyzed is the winner or loser o the set currently being analyzed. Each of the points analyzed will have two rows; one will correspond to the data of the winner of the set and another to the loser's data. Contrary to what happened in the previous variable, the first row does not have to correspond to the winner of the match nor the second row to the loser.

1	Winner of the set
0	Loser of the set

2.6 **Game score:** this variable indicates the score in the game before the player hits the shuttlecock, taking into account that the score of the player who starts the game serving is scored first.

00	0/0
10	1/0
01	0/1
…	…

2.7 Winner or loser of the analyzed point: this variable indicates the player who ends up winning the analyzed point. It does not have to correspond with the winner and loser of the match.

1	Winner of the point
0	Loser of the point

3. VARIABLES RELATED TO THE GAME

The variables related to the game register a large number of technical and tactical aspects which occur from the moment the shuttlecock is put into play by the server until the end of the point. Some of these aspects correspond to the different types of strokes, stroke sequences, hitting areas, together with the effectiveness.

3.1 Stroke sequence: temporal sequence or chronological order of the strokes which occur in the dispute of a point between two players. The aim is to classify the types of hitting in a temporal order associated with possible technical and tactical imbalances.

0	Serve error
1	Serve
2	2nd stroke
3	3rd stroke
…	…
99	Penultimate stroke of the point
100	Last stroke of the point

0 Serve error: it is register only when a serve error occurs.
1 Serve: stroke which corresponds to the serve.
2, 3 Second stroke, third stroke: temporarily ordered squence of strokes which occurs after the serve return and ends after the penultimate hit of the disputed point.
99 Penultimate stroke of the point: stroke which occurs before the last stroke made in the dispute of a point between two players.
100 Last stroke of the point: last stroke which occurs in the dispute of a point between two players.

3.2 Point duration time: time duration of the point sequence (minutes/seconds).

3.3 **<u>Kind of technical and tactical stroke:</u>** the criteria of the Spanish Badminton Federation (FESBA) have been used to define each type of the stroke.

11	Right serve
12	Reverse serve
21	Clear from right to high hand
22	Clear from right to médium height
23	Clear from right to low hand
24	Clear from left to high hand
25	Clear from left to medium height
26	Clear from left to low hand
31	Right drop
32	Left drop
41	Right smash
42	Left smash
43	Smash in jump
51	Drive from right to high hand
52	Drive from right to medium height
53	Drive from left to high hand
54	Drive from left to medium height
61	Net drop from right to medium height
62	Net drop from right to low hand
63	Net drop from left to medium height
64	Net drop from left to low hand
71	Lob from right to medium height
72	Lob from right to low hand
73	Lob from left to medium height
74	Lob from left to low hand
81	Right brush
82	Left brush
91	Right kill
92	Left kill
101	Right push
102	Left push

1…SERVE: type of stroke which is made from one of the serve squares to the diagonally opposite square (according to the rules). Generally it is executed in sucha a way that at the moment of impact the shuttlecock is below the waist of the server, therefore being a defensive stroke due to the trajectory that it describes. As with most badminton strokes, the main purpose of the serve is to score the point with this single stroke. This objective, which in principle is evident, is hampered by the same regulation since it forces us to make a bottom-up hit, as well as the great possibilities of the opponent to return the shuttlecock.

11 Right serve: action of the serve, hitting with the dominant plane of the racket.
12 Reverse serve: action of the serve, hitting with the other side of the dominant plane of the racket.

2…CLEAR: defensive strike which is preferably executed from the bottom of the court, and whose trajectory goes from the end of the court to the bottom of the opponent's field. The purpose of this stroke is to move the opponent to the end of the court.
21 Clear from right to high hand: action of the clear, hitting with the dominant plane of the racket over the shoulder.
22 Clear from right to medium height: action of the clear, hitting with the dominant plane of the racket between the soulder and the waist.
23 Clear from right to low hand: action of the clear, hitting with the dominant plane of the racket below the waist.
24 Clear from left to high hand: action of the clear, hitting with the other side of the dominant plane of the racket over the shoulder.
25 Clearfrom left to medium height: action of the clear, hitting with the other side of the dominant plane of the racket between the soulder and the waist.
26 Clear from left to low hand: action of the clear, hitting with the other side of the dominant plane of the racket below the waist.

3…DROP: offensive precisión strike which is mainly executed from the bottom of the court in a high hand, the trajectory is descending to pass as close to the net and fall approximately on the serve line. This stroke can also be made from the center of the court to the net.
31 Right drop: action of the drop, hitting with the dominant plane of the racket.
32 Left drop: action of the drop, hitting with the other side of the dominant plane of the racket.

4…SMASH: quintessential offensive stroke. It is executed from any part of the court and always in a high hand. The trajectory is descending, being able to fall halfway or bottom of it and characterized by its speed and trajectory.
41 Right smash: action of the smash, hitting with the dominant plane of the racket.
42 Left smash: action of the smash, hitting with the other side of the dominant plane of the racket.
43 Smash in jump: it is one of the most spectacular and effective actions in badminton and is achieved by performing the action of the smash while the player is in suspension.

5…DRIVE: offensive strike whose purpose is the shuttlecock pass as close to the net as possible, quickly and parallel to the ground. It aims to cause a weak return fromthe opponent, in addition to maintaining the attack in any situation. Its execution is carried out at an approximate height between the head and the waist, keeping the shuttlecock in a parallel-descending trajectory to the ground.

51 Drive from right to high hand: action of the drive, hitting with the dominant plane of the racket over the shoulder.
52 Drive from right to medium height: action of the drive, hitting with the dominant plane of the racket between the soulder and the waist.
53 Drive from left to high hand: action of the drive, hitting with the other side of the dominant plane of the racket over the shoulder.
54 Drive from left to medium height: action of the clear, hitting with the other side of the dominant plane of the racket between the soulder and the waist.

6…NET DROP: offensive stroke which is executed from a position close to the net, the shuttlecock describing a trajectory as close to the net as possible both in height and in distance.
61 Net drop from right to medium height: action of the drop, hitting with the dominant plane of the racket between the soulder and the waist.
62 Net drop from right to low hand: action of the drop, hitting with the dominant plane of the racket below the waist.
63 Net drop from left to medium height: action of the drop, hitting with the other side of the dominant plane of the racket between the soulder and the waist.
64 Net drop from left to low hand: action of the drop, hitting with the other side of the dominant plane of the racket below the waist.

7…LOB: defensive stroke which is executed near the net and is directed towards the bottom of the court.
71 Lob from right to medium height: action of the lob, hitting with the dominant plane of the racket between the soulder and the waist.
72 Lob from right to low hand: action of the lob, hitting with the dominant plane of the racket below the waist.
73 Lob from left to medium height: action of the lob, hitting with the other side of the dominant plane of the racket between the soulder and the waist.
74 Lob from left to low hand: action of the lob, hitting with the other side of the dominant plane of the racket below the waist.

8…BRUSH: offensive high-hand stroke which is executed very close to the net with a lateral racket trajectory, avoiding contact with the net.
81 Right brush: action of the brush, hitting with the dominant plane of the racket.
82 Left brush: action of the brush, hitting with the other side of the dominant plane of the racket.

9…KILL: specific high-hand offensive stroke which is executed in positions very close to the net with a descending trajectory, used to end the play.
91 Right kill: action of the kill, hitting with the dominant plane of the racket.
92 Left kill: action of the push, hitting with the other side of the dominant plane of the racket.

10…PUSH: offensive high-hand stroke made very close to the net where the shuttlecock is pushed to fall before the service line of the opposite field.
101 Right push: action of the push, hitting with the dominant plane of the racket.
102 Left push: action of the push, hitting with the other side of the dominant plane of the racket.

3.4 Trajectory: shuttlecock trajectory.

1	Paralell
2	Cross

3.5 Tactical intentionality: player's tactical intent.

1	Offensive
2	Defensive

3.6 Hitting area: position in which the player's legs are when the shuttlecock is hit. There will be occasions when legs are between two zones, in that case the zone with the highest proportion of body at the momento of impact will be considered.

11	Inside the court, serve and background zone, in the left area
12	Inside the court, serve and background zone, in the central area
13	Inside the court, serve and background zone, in the central area
14	Inside the court, serve and background zone, in the right area
21	Serve zone, in the left area
22	Serve zone, in the central area
23	Serve zone, in the central area
24	Serve zone, in the right area
31	Near the net, in the left area
32	Near the net, in the central area
33	Near the net, in the central area
34	Near the net, in the right area

**The badminton court will be divided into 12 zones, each and every one of the same size:*

11 Inside the court, serve and background zone, in the left area: the shuttlecock is hit in the far left area of the court as the player looks at the net.
12 Inside the court, serve and background zone, in the central area: the shuttlecock is hit in the bottom area located on the left side closest to the central area of the court as the player looks at the net.
13 Inside the court, serve and background zone, in the central area: the shuttlecock is hit in the bottom area located on the right side closest to the central area of the court as the player looks at the net.
14 Inside the court, serve and background zone, in the right area: the shuttlecock is hit in the far right area of the court as the player looks at the net.
21 Serve zone, in the left area: the shuttlecock is hit in the half area located to the left side of the court as the player looks at the net.

22 Serve zone, in the central area: the shuttlecock is hit in the middle zone located on the left side closest to the central zone of the court as the player looks at the net.
23 Serve zone, in the central area: the shuttlecock is hit in the middle zone located on the right side closest to the central zone of the court as the player looks at the net.
24 Serve zone, in the right area: the shuttlecock is hit in the half area located to the right side of the court as the player looks at the net.
31 Near the net, in the left area: the shuttlecock is hit in the closest area to the net located to the left side of the court as the player looks at the net.
32 Near the net, in the central area: the shuttlecock is hit in the closest area to the net located on the left side closest to the central area of the court as the player looks at the net.
33 Near the net, in the central area: the shuttlecock is hit in the closest area to the net located on the right side closest to the central area of the court as the player looks at the net.
34 Near the net, in the right area: the shuttlecock is hit in the closest area to the net located to the right side of the court as the player looks at the net.

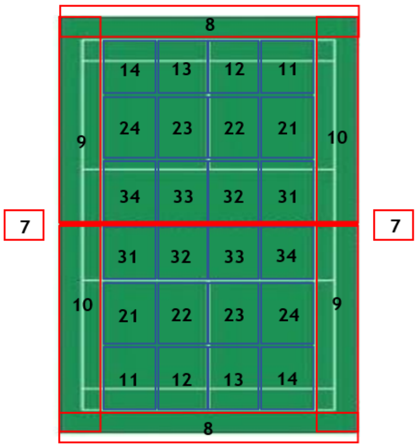

Figure 1. *Player hitting areas.*

3.7 **<u>Stroke efectiveness:</u>** result of the stroke made by the player in terms of effectiveness.

1	Winner
2	Total continuity
3	Partial continuity
4	Error

1 Winner: stroke made by a player, obtaining a point directly, without the opponent hit the shuttlecock.
2 Total Continuity: transitional stroke made by a player, sending the shuttlecock to the opposite field, continuing the opponent the point (without failure).
3 Partial Continuity*:* stroke made by a player, sending the shuttlecock to the opposite field, causing the opponent hit the shuttlecock, sending it outside the limits of the court or to the net (with failure).
4 Error: stroke made by a player, sending the shuttlecock out of the limits of the court, to the net, to the ceiling or to the own field.
